# Influence of environmental factors on biodegradation of quinalphos by *Bacillus thuringiensis*

**DOI:** 10.1186/s12302-017-0109-x

**Published:** 2017-03-06

**Authors:** Venkata Subba Reddy Gangireddygari, Praveen Kumar Kalva, Khayalethu Ntushelo, Manjunatha Bangeppagari, Arnaud Djami Tchatchou, Rajasekhar Reddy Bontha

**Affiliations:** 1 Department of Microbiology, Sri Krishnadevaraya University, Anantapuramu, Andhra Pradesh 515 003 India; 2 College of Agriculture & Environmental Sciences, Department of Agriculture & Animal Health, Florida Science Campus, Corner Christiaan De Wet and Pioneer Avenue, Florida, University of South Africa, Johannesburg, Gauteng, 1710 South Africa; 3 Department of Life Sciences, Universidad de las Fuerzas Armadas-ESPE, Sangolqui, Quito, Ecuador

**Keywords:** Quinalphos, 16S rRNA gene, *B. thuringiensis*, Generation time

## Abstract

**Background:**

The extensive and intensive uses of organophosphorus insecticide—quinalphos in agriculture, pose a health hazard to animals, humans, and environment because of its persistence in the soil and crops. However, there is no much information available on the biodegradation of quinalphos by the soil micro-organisms, which play a significant role in detoxifying pesticides in the environment; so research is initiated in biodegradation of quinalphos.

**Results:**

A soil bacterium strain, capable of utilizing quinalphos as its sole source of carbon and energy, was isolated from soil via the enrichment method on minimal salts medium (MSM). On the basis of morphological, biochemical and 16S rRNA gene sequence analysis, the bacterium was identified as to be* Bacillus thuringiensis*.* Bacillus thuringiensis* grew on quinalphos with a generation time of 28.38 min or 0.473 h in logarithmic phase. Maximum degradation of quinalphos was observed with an inoculum of 1.0 OD, an optimum pH (6.5–7.5), and an optimum temperature of 35–37 °C. Among the additional carbon and nitrogen sources, the carbon source—sodium acetate and nitrogen source—a yeast extract marginally improved the rate of degradation of quinalphos.

**Conclusions:**

Display of degradation of quinalphos by* B*.* thuringiensis* in liquid culture in the present study indicates the potential of the culture for decontamination of quinalphos in polluted environment sites.

## Background

Organophosphate (OP) compounds are part of the most common chemical classes used in the protection of crop and livestock and in the control of diseases transmitted through vectors and account for an estimated 34% of worldwide insecticide sales [1]. In India, usage of OPs has also gradually been increasing with a consistent decline in application of organochlorines and currently makes up 27% of the total sales of pesticides [2–4]. Andhra Pradesh is the biggest user of crop protection chemicals in India and uses 20% of the total pesticides in the country [5]. Among OPs, quinalphos (QP: *O,O*-diethyl *O*-quinoxalin-2-yl phospharothioate) is used widely in agriculture in Andhra Pradesh because of its effective control of all pests over different crops, which is reflected by the 5% of total sales of pesticides registered against quinalphos [5]. Quinalphos is a synthetic OP, non-systemic, broad spectrum insecticide, and acaricide extensively used in India owing to its action on inhibition of acetylcholinesterase in target pests [6, 7]. Being ranked as moderately hazardous by the World Health Organization (WHO) and classified as a yellow label (highly toxic) pesticide in India, quinalphos is either banned or restricted in its usage in most of the nations [8]. Nevertheless, quinalphos is still being used to treat the following crops: wheat, rice, groundnut, cotton, sugarcane, coffee and other ornamental crops. Only 1% of the pesticides applied make contact with the target pest, while the remaining 99% of the pesticide drifts into the environment contaminating soil, water and biota [9, 10]. Accidental spills/leaks occurring during transport and storage of industrial materials and agricultural chemicals have polluted areas that were never intended as sites for waste disposal. Thus, soil and water bodies serve as the ultimate receptacle/reservoir for all kinds of pesticides regardless of whether they are applied intentionally or unintentionally.

The extensive usage of quinalphos in agriculture poses a health hazard because of severe inhibition of acetylcholinesterase (AChE) in non-target organisms by quinalphos [11–15], an adverse influence on blood and brain esterase activity in chickens [16] and fertility efficiency in adult male rats [17] by quinalphos. Exposure of non-target organisms to quinalphos in the environment depends on the extent of persistence of quinalphos in natural resources which is, in turn, controlled by factors—abiotic and biotic. Influence of abiotic factors such as sun light, pH and TiO_2_ on degradation of quinalphos in natural resources such as soil and water was examined [18–22]. Relatively, less attention was paid on biotic factors involved in the fate of quinalphos in natural resources [23]. A definite participation of factors, in particular biotic factors, in the degradation of quinalphos in natural resources such as soil and water can only be demonstrated with the isolation of biotic agents with degradation traits from natural resources. Isolation of *Ochrobactrum* sp. strain HZM with biodegradation of quinalphos from pesticide-contaminated samples has been recently reported [23]. This organism degraded quinalphos by hydrolysis. Strains of *Bacillus thuringiensis* appeared to be biotic agents for degradation of fipronil and a wide range of pyrethroids in sugarcane fields [24] and an activated sludge [25]. In view of less understanding of biotic factors in quinalphos degradation, the current study is aimed at isolating bacterial species capable of degrading quinalphos from soil samples collected from horticultural fields and hitting the potential bacterium for assessment of various environmental factors on biodegradation of quinalphos in liquid culture conditions.

## Methods

### Soils

Soil samples [organic matter (%)—0.45; nitrogen (%)—1.62; pH—7.86] were collected from a horticultural field in a semi-arid zone at Honegal close to Chikkaballapur, Karnataka, India.

### Chemicals

Quinalphos of a technical grade was purchased from Sigma-Aldrich (99.2% purity). This quinalphos was used for bacterial growth as a sole source of carbon and energy. All other chemicals and solvents used in the study were of an analytical reagent grade/HPLC grade and purchased from Sigma-Aldrich.

### Culture medium and selective enrichment method

The composition of the mineral salt medium (MSM) was as follows (g L^−1^): 1.5 NH_4_NO_3_, 1.5 K_2_HPO_4_·3H_2_O, 0.2 MgSO_4_·7H_2_O, 1.0 NaCl and 1 mL of trace element stock solution. The trace element stock solution contained the following (g L^−1^): 2.0 CaCl_2_·2H_2_O, 0.2 MnSO_4_·4H_2_O, 0.1 CuSO_4_·2H_2_O, 0.2 ZnSO_4_·H_2_O, 0.02 FeSO_4_·7H_2_O, 0.09 CoCl_2_·6H_2_O, 0.12 Na_2_MoO_4_·2H_2_O and 0.006 H_3_BO_3_.

For selective enrichment, 5-g samples of soil were incubated in MSM spiked with quinalphos of the technical grade at 20 µg mL^−1^ of MSM in a 250-mL Erlenmeyer flask in an orbital shaker (Orbitek LE-IL Model) at 37 °C and 175 rpm. After 10 days of incubation, a 5-mL portion of the culture was transferred to a fresh medium fortified with increasing concentrations of quinalphos up to 200 µg mL^−1^ in Erlenmeyer flasks and the flasks were incubated for an additional 10 days. After five more transfers, the culture was purified by serial dilution and streak plating onto solidified MSM containing 20 µg mL^−1^ of quinalphos. Finally, a pure bacterial strain was obtained and designated as OP1.

## Identification and characterization of the bacterial isolate

### Morphological, physiological and biochemical characterization

Morphological observations of bacterial isolate were made with an optical compound microscope. Physiological and biochemical properties of the isolate were determined by the procedures as described in Bergey’s Manual of Determinative Bacteriology [26].

### 16S rRNA gene sequencing and phylogenetic tree analysis

Amplification of the 16S rRNA gene in genomic DNA, extracted from the potential bacterial isolate (OP1) in a standard phenolic extraction procedure [27], was performed with the universal conserved sequence as primers—16 forward primer sequence, 5′-AGACTCAGGTTTGATCCTGG-3′, and 16 reverse primer sequence, 5′-ACGGCTACCTTGTTACGACTT-3′. The phylogenetic analysis was based on a 16S rRNA gene sequence as described by Qin et al. [28]. Comparison of the determined sequence with those in the GenBank/EMBL database was made using the online tool BLAST programme [29]. Sequences of the OP1 and closely related bacterial spp. were collected and aligned. A neighbour-joining and maximum-likelihood tree was constructed using the Robust Phylogenetic tree online tool [30, 31] to establish the phylogenetic relationship.

## Measurement of bacterial growth kinetics on quinalphos

For the preparation of the inoculum, the bacterial isolate OP1 was grown overnight in 50 mL of MSM amended with 20 ppm of quinalphos per mL of MSM and yeast extract (0.1%) on an orbital shaker at 175 rpm at 37 °C. Bacterial cells in the overnight grown culture were harvested aseptically (8000×*g*, 15 min, 4 °C) and thoroughly washed with MSM and suspended in sterile MSM to get a suspension with the desired OD. For the growth of the bacterial isolate on quinalphos, 50 mL of sterile MSM, spiked with quinalphos at a concentration of 20 µg mL^−1^, was dispensed into sterile 250-mL Erlenmeyer flasks. After inoculation with the bacterial culture to the final OD of 1.0/mL of MSM, the flasks were incubated on an orbital shaker at 175 rpm at 37 °C. Uninoculated flasks with the fortified medium served as the control. Five-millilitre aliquots from the growing culture broth were withdrawn at 6-h intervals for measurement of turbidity/growth at wavelength of 600 nm in a UV–visible spectrophotometer (Chemito-UV-2600). The total number of viable bacterial colony-forming units in the culture broth was determined by a serial dilution method on nutrient agar medium plates. The specific growth rate of bacterial sp. OP1 was calculated in the logarithmic phase.

## Biodegradation of quinalphos

Experiments on biodegradation of quinalphos by the bacterial isolate was undertaken in 250-mL Erlenmeyer flasks in the same manner as done for the growth experiments as mentioned earlier “Measurement of bacterial growth kinetics on quinalphos” section. Flasks containing quinalphos in MSM without inoculum served as controls. At regular intervals of 48 h, 10 mL of culture broth was aseptically withdrawn from the flasks for growth measurements and residue analysis. The culture broth from both uninoculated and inoculated flasks was processed for residue analysis and spun at 8000×*g* for 15 min in a refrigerated centrifuge (REMI, C24 BL, Hyderabad). The supernatants collected were extracted with dichloromethane with an equal volume of supernatant; this was repeated three times with fresh lots of dichloromethane. The extracts were pooled together, dried over anhydrous sodium sulphate, filtered and allowed to dry at room temperature. The dried residue was dissolved in methanol for UFLC analysis.

### Factors influencing biodegradation of quinalphos

In order to assess the effect of various factors on the degradation of quinalphos by OP1, appropriate modifications in the supplementation of additional nutrients to MSM and the growth conditions of the bacterial culture on quinalphos were made. For this purpose, MSM was spiked with 20 mg L^−1^  of quinalphos and distributed into 250-mL flasks (100 mL per flask). The flasks were supplemented with an additional carbon source, (glucose or sodium acetate), or additional nitrogen sources, NH_4_Cl, (NH_4_)_2_SO_4_, urea or yeast extract to a final concentration of 0.01% (w/v). Flasks were inoculated with the bacterial suspension to get an initial OD of 1.0, and flasks devoid of inoculum were maintained as controls. These flasks were incubated at 37 °C and shaken at 175 rpm in an orbital shaker; samples were collected at 48-h intervals; and the culture broth was extracted with dichloromethane solvent for residue analysis. The influence of the concentration of quinalphos on its degradation was assessed by growing the bacterial isolate on quinalphos in MSM at different concentrations (20–200 ppm) of quinalphos. In another experiment, flasks containing MSM (pH 7.5) were supplemented with 20 mg L^−1^ of quinalphos inoculated with the bacterial cell suspension to an initial OD of 1.0 and incubated in a shaker at 175 rpm at different temperatures of 30–45 °C to study the influence of temperature on the degradation of quinalphos. In order to study the effect of pH on quinalphos degradation, OP1 was cultured as described above and only the pH was varied from a pH of 5.5–8.5.

### Quinalphos residue analysis by ultra-fast liquid chromatography (UFLC)

The residue of quinalphos extracted from the different experiments was dissolved in methanol and analysed by UFLC-LC 20 AD (Shimadzu, Japan) equipped with a ternary gradient pump, programmable variable-wavelength PDA detector, column oven, and electric sample valve and ODS-2, C_18_, reverse-phase column (4.6 × 250 mm × 5 μm). The quinalphos residue analysis was conducted using an isocratic mobile phase of methanol. The sample injection volume was 20 µL; the mobile phase was programmed at a flow rate of 1 mL min^−1^; and quinalphos was detected at 254 nm wavelength under these operating conditions with a retention time of 1.859 min.

## Statistical analysis

All parameters (carbon source, nitrogen source, size of inoculum, concentration of quinalphos, pH and temperature) were compared using a one-way ANOVA analysis. All were tested at *P* < 0.05 significance level and the Duncan multiple range test was used for separation between treatment means. Statistica v.10, StatSoft (USA) was used for all statistical analysis.

## Results and discussion

### Identification and characterization of the bacterial isolate

The bacterial strain (OP1) was isolated from a horticultural field by the selective enrichment method. The OP was identified according to the classification scheme outlined by Bergey’s Manual of Determinative Bacteriology [26]. The cell morphology for OP1 was analysed by compound microscopy and observed to display morphological characteristics consistent with the Gram-positive reaction; the colonies were rod-shaped, circular with a crenate margin. In addition, various biochemical tests were performed and recorded as Indole test—negative; Methyl red test—negative; VP test—negative; Citrate utilization test—positive; glucose and lactose fermentation tests—positive; Urease activity—positive; Catalase activity—positive; Nitrate-reductase activity—positive; Starch hydrolysis—positive; Casein hydrolysis—negative; Gelatin liquefaction—negative. Based on these morphological and biochemical characteristics, the strain OP1 is homologous with *Bacillus* sp.

**Fig. 1:**
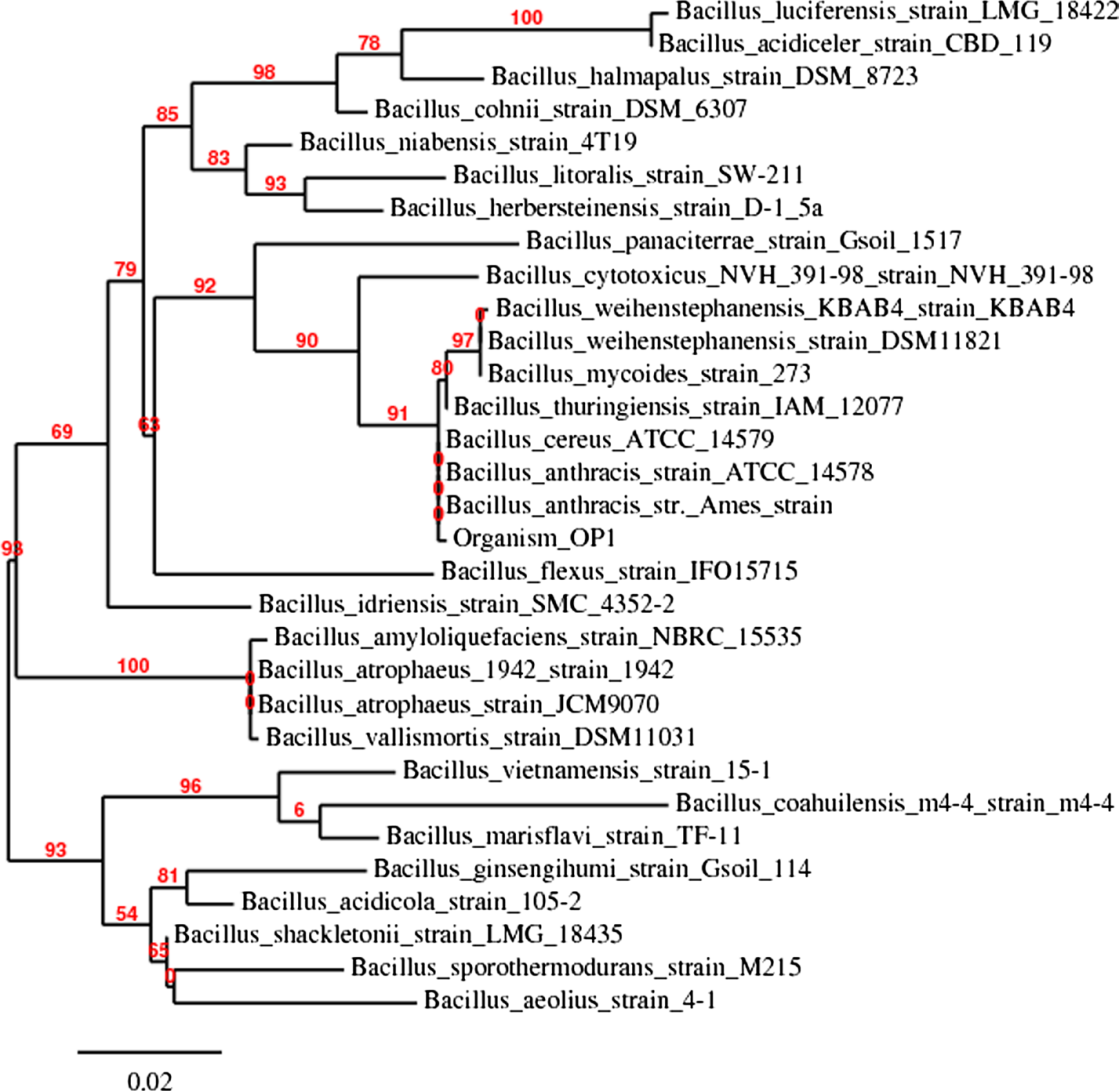
Phylogenetic tree based on the 16S rRNA gene sequences of strain OP1. Robust phylogenetic tree showing the phylogenetic relationship between strain OP1 and related species based on the 16S rRNA gene sequences. Bootstrap values obtained with 1000 repetitions were indicted as percentages at all branches

The 16S rRNA gene sequence was analysed with the Robust Phylogenetic tree online tool [30, 31], and the neighbour-joining dendrogram was constructed (Fig. 1). Based on the dendrogram, morphological and biochemical characteristics, OP1 showed 91% homology with *Bacillus thuringiensis* strain IAM 120177 and as a result was tentatively identified as *B. thuringiensis.* The nucleotide sequence encoding the 16S rRNA gene of *B. thuringiensis* (931 bases) was deposited in the GenBank database with the accession number of KC577853.

As the 16S rRNA gene sequence is a proven molecular and taxonomic tool used for the identificat'ion of bacteria isolated from the environment [23, 32–36], the same approach was adopted for the identification of bacterial isolate in the current study. Similarly, strains of *Bacillus thuringiensis* with capacity to degrade insecticides—cyhalothrin (http://www.nature.com/articles/srep0874) and fipronil [24, 25] were isolated from an activated sludge and sugarcane growing fields, respectively.

### Growth rate of *Bacillus thuringiensis* on quinalphos

A bacterial culture—*B. thuringiensis*—was grown on quinalphos at concentration of 20 µg mL^−1^ in MSM, and viable cell counts of *B. thuringiensis* were enumerated at regular intervals. The results of the viable cell counts in terms of colony-forming units (CFU) are represented in Table 1. The initial viable cell count with 26 × 10^8^ CFU mL^−1^ of *B. thuringiensis* in the medium at the time of inoculation rose to 190 × 10^8^ mL^−1^ within 6 h of incubation. During the log phase, the bacterial growth rate and generation time were calculated as per the equation

$$K = { \log }N_{\text{t}} - { \log }N_{\text{o}} /{\text{log 2}} \times t,$$

where *N*_t_ and *N*_o_ are bacterial populations at *t* and 0 times, respectively. The generation time of *B. thuringiensis* in the log phase was 28.38 min or 0.473 h.

**Table 1:** Growth of *Bacillus thuringiensis* on quinalphos

Incubation time in h	*Bacillus thuringiensis* CFU/mL
0	26 × 10^8^
6	190 × 10^8^
12	570 × 10^8^
18	600 × 10^8^
24	95 × 10^8^

Quinalphos was included in MSM as the sole source of carbon and energy for the cultivation of a bacterial culture in the current study. The proliferation of bacterial cells on quinalphos occurred in MSM up to 18 h as reflected by an increase in the viable cell count (Table 1). This indicates the use of quinalphos by bacterial culture as a sole source of carbon.

Under the conditions used in the current study, *B. thuringiensis* grew more rapidly with increase in cell number and a shorter generation time. Proliferation of organisms is only possible with utilization of OP insecticides as carbon and energy source. Similarly, utilization of quinalphos at 2 mmol L^−1^ as a sole source of carbon and energy by *Ochrobactrum* sp. strain HZM and attainment of maximum growth (OD_600_—0.8) within 6 days of incubation was reported [23]. The growth of bacteria—*Pseudomans* sp. and *Serratia* sp.—on diazinon at 50 mg L^−1^ in MSM was the most effective, attaining a maximum OD_660_ within 6–10 days [37]. The growth of *Pseudomonas putida* epI on ethoprophos appeared to be a logarithmic mode between 5 and 35 h of incubation [38]. During this period, the viable cell count in the culture increased from 10^4^ to 10^6^ CFU mL^−1^. There was a rapid increase in the OD_600_ value up to 0.9 of the culture of *Paracoccus* sp. for 2 days with the utilization of 50 µg mL^−1^ of chlorpyrifos [39]. *Alcaligenes* sp. JAS1 could grow rapidly on chlorpyrifos at 300 mg L^−1^ for 5 days and exhibited a high growth rate [36].

## Biodegradation of quinalphos by *Bacillus thuringiensis*

Variable factors such as additional carbon and nitrogen sources, the size of inoculum density, medium pH, temperature and concentration of quinalphos were examined to optimize the biodegradation of quinalphos by *B. thuringiensis.*

### Influence of additional carbon source on biodegradation of quinalphos

**Fig. 2:**
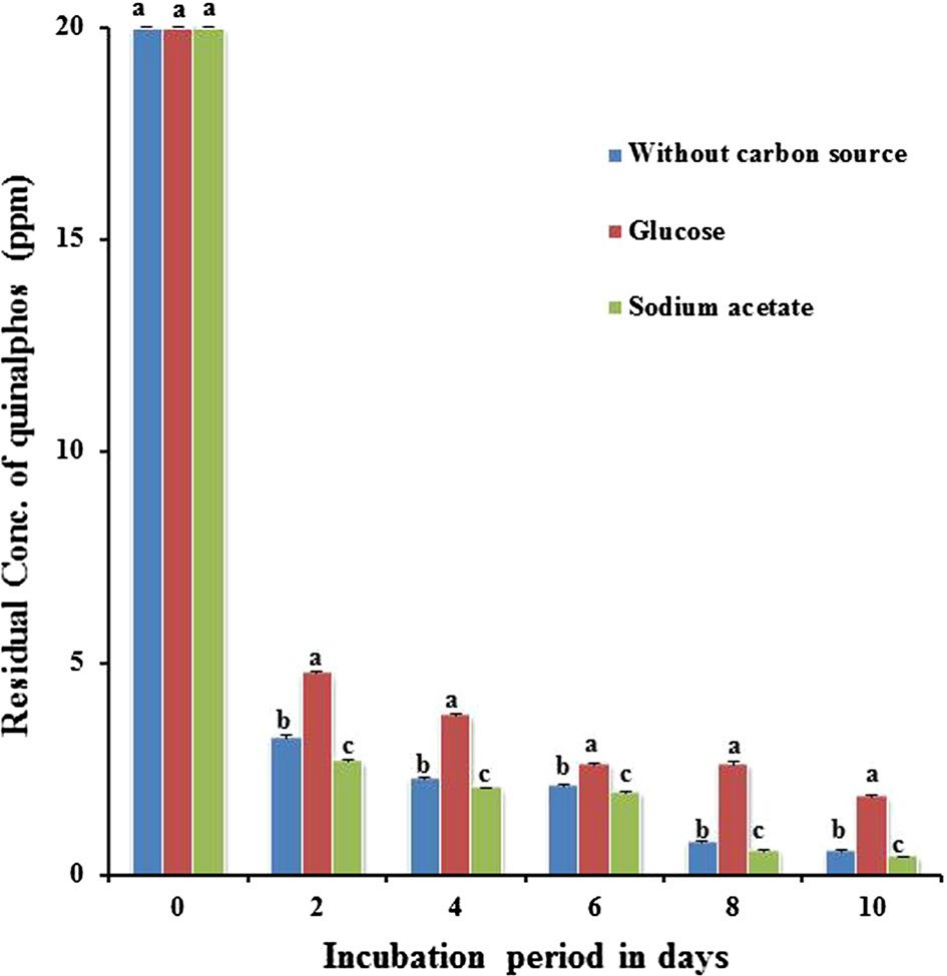
Influence of additional carbon source on biodegradation of quinalphos. Values represented by *bars* with the same letter (*a, b, c*) are not statistically significant at *P* < 0.05

In order to find out the influence of supplementation with an additional carbon source on biodegradation of quinalphos, the bacterial strain *B. thuringiensis* was grown on quinalphos in MSM with/without glucose or sodium acetate at 0.01%. The concentration of quinalphos residues in a culture broth in the presence or absence of additional carbon was quantified, and the results are presented in Fig. 2.

Disappearance of quinalphos occurred to the extent of 84–86% with the culture of *B. thuringiensis* grown in the presence of sodium acetate or absence of any additional carbon within 2 days of incubation. However, degradation of quinalphos was slower in the cultures of the same bacterium grown with the amendment of glucose as reflected by the recovery of 24% initially added quinalphos at the end of 2-day incubation.

Influence of additional carbon source on degradation of OP compounds varies from one organism to another. For instance, a provision of additional carbon source to *Paracoccus* strain TRP [39] and *Enterobacter* strain B-14 [40] led to a lag phase followed by an accelerated degradation of chlorpyrifos. The degradation of ethoprophos by *Pseudomonas putida* epI was not markedly influenced by the presence of a supplementary carbon source [38]. Ethoprophos degradation by *P. putida* epII was slower in the presence of glucose or succinate. On the other hand, glucose enhanced the degradation rate of *Bacillus pumilus* C2A1 as it completely degraded the chlorpyrifos within 3 days of incubation. However, results of the current study revealed that supplementation of carbon had a different response to the degradation of quinalphos by the *B. thuringiensis*. The disappearance of quinalphos by *B. thuringiensis* in the presence of sodium acetate was marginally improved. This might be due to the effect of sodium acetate on the growth of the bacterium.

### Influence of an additional nitrogen source on the biodegradation of quinalphos

**Fig. 3:**
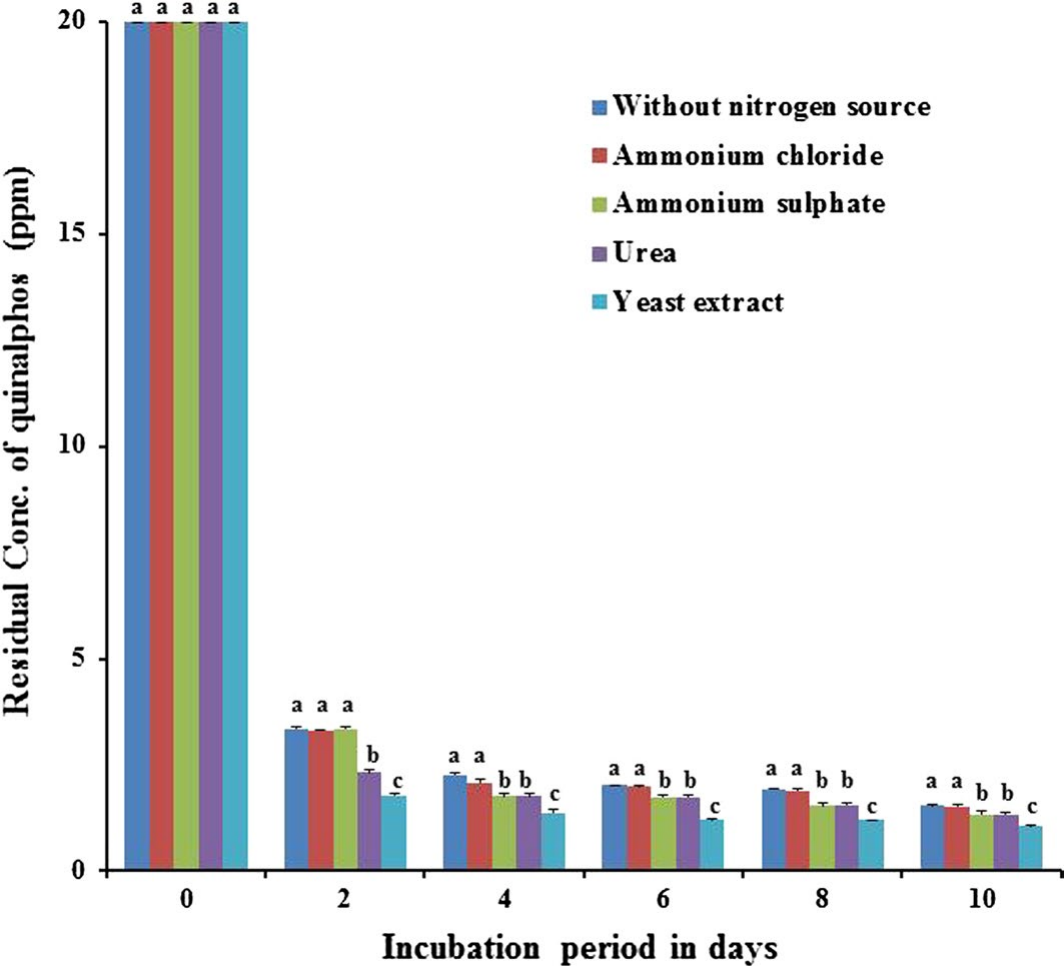
Influence of additional nitrogen source on biodegradation of quinalphos. values represented by *bars* with the same letter (*a, b, c*) are not statistically significant at *P* < 0.05

The effect of various nitrogen sources, such as inorganic sources, ammonium chloride or ammonium sulphate, and organic sources, urea and yeast extract, was examined on the biodegradation of quinalphos by the bacterial isolate *B. thuringiensis.* The extent of degradation of quinalphos by *B. thuringiensis* without an additional nitrogen source (control) and with the addition of nitrogen sources—ammonium chloride, ammonium sulphate, urea and yeast extract—occurred in the range of 83.66–88.4% as against 91.1% in the presence of the yeast extract at the end of 2nd day incubation (Fig. 3).

Quinalphos contains nitrogen atoms in its structure. A provision of another nitrogen source in the medium may cause a reduction in the degradation and utilization of nitrogenous organophosphates as a nitrogen source. The addition of yeast extract and nutrient broth to MSM enhanced the degradation of non-nitrogenous OP pesticide, chlorpyrifos, by the *Bacillus pumilus* strain [41]. In the current study, supplementation of nitrogen, except for the yeast extract, did not improve degradation of quinalphos.

### Influence of inoculum density on the biodegradation of quinalphos

**Fig. 4:**
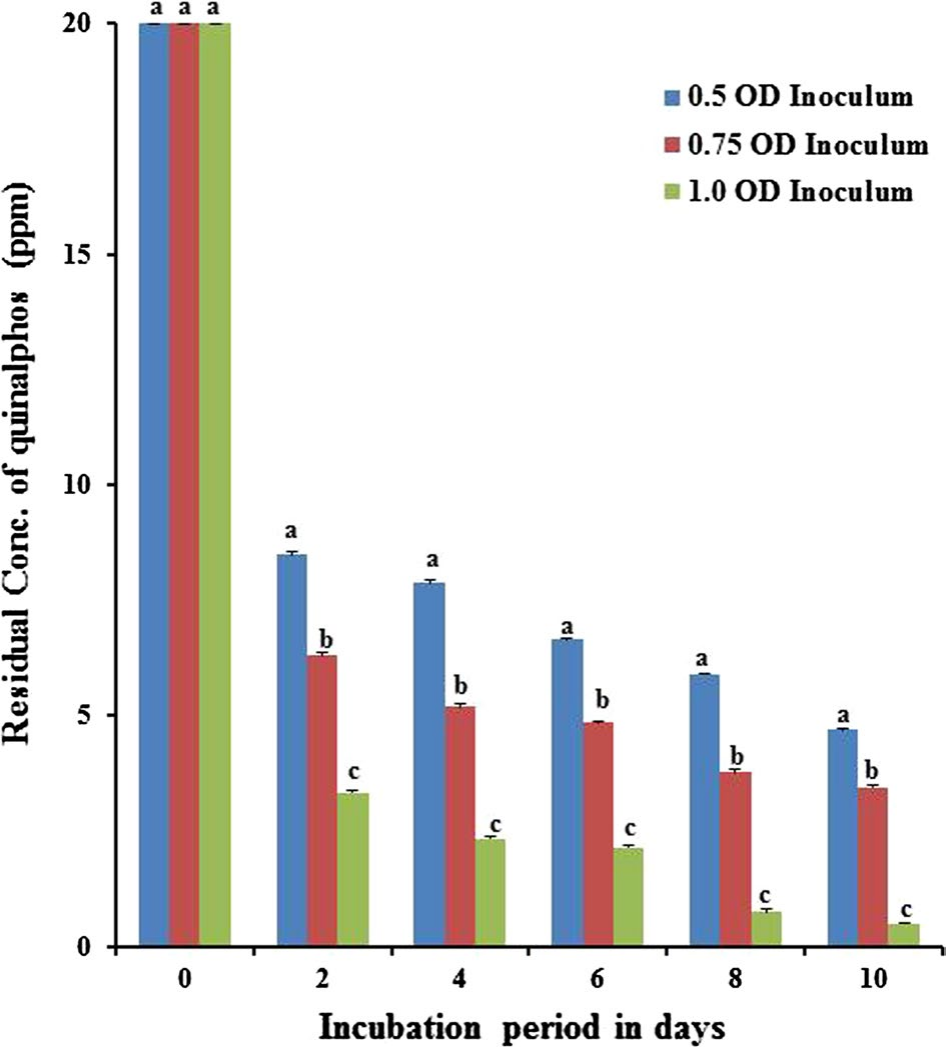
Influence of inoculum density on biodegradation of quinalphos. Values represented by *bars* with the same letter (*a, b, c*) are not statistically significant at *P* < 0.05

In order to find out the optimal size of inoculum density for degradation of quinalphos, fortified MSM was tested with the three different sizes of inoculum densities of the potential bacterial isolate *B. thuringiensis*. The cell suspension of the culture was diluted to different cell densities and added to MSM fortified with quinalphos to provide final bacterial cell densities of 0.5, 0.75 and 1.0 OD at *A*_600_ in inoculated medium at day 0. The culture was grown at 30 °C in an orbital shaker and the parent compound quinalphos; the residue in the medium was quantified at 2-day intervals (Fig. 4). Quinalphos degradation occurred to the extent of 57, 68 and 83% of the initially added amount in a 2-day old culture of *B. thuringiensis* inoculated with a density of 0.5, 0.75 and 1.0 OD, respectively. The highest cell density, i.e. 1.0 OD, used in the current study supported the maximum degradation of quinalphos by *B. thuringiensis*. However, quinalphos degradation occurred to a lesser extent with lower inoculum densities of 0.5 and 0.75 OD of *B. thuringiensis*. It is clear from the results of this study that 1.0 OD size of inoculum density favoured the degradation of quinalphos by *B. thuringiensis*. Growth of culture of *B. thuringiensis* with a smaller inoculum density took longer lag phase for commencement of degradation of quinalphos. Similarly, both strains *Pseudomonas putida* epI and epII totally degraded ethoprophos at 10 mg L^−1^ level in MSM within 30–48, 80–98 and 130–150 h after inoculation with different densities of 6 × 10^6^, 6 × 10^4^, 6 × 10^2^ cells mL^−1^, respectively [38]. Anwar et al. [41] assessed the influence of inoculum densities on the degradation of chlorpyrifos by the *Bacillus pumilus* C2A1 strain. In cultures of *B. pumilus* C2A1 grown with lower inoculum densities, i.e. 10^5^ and 10^7^ CFU mL^−1^, there were longer lag periods as degradation of chlorpyrifos started after 1 and 2 days of incubation and resulted in 50 and 72% degradation of pesticide, respectively, after 5 days of incubation. Chlorpyrifos degradation started rapidly apparently without a lag phase in the same culture grown with 10^9^ CFU mL^−1^, and more than 80% degradation was deliberated after 5 days of incubation. A possible explanation may be that microorganisms need an adaptation period to produce a considerable population size with the secretion of necessary degradative enzymes to cause noticeable degradation. This may be the reason for the prolonged lag phase when grown in a medium with a low density of cells in degradation of chlorpyrifos [42].

### Influence of concentration of quinalphos on its biodegradation

In order to assess the degradation capacity of quinalphos by *B. thuringiensis* culture, MSM was fortified with quinalphos at different concentrations within a range of 20–200 mg L^−1^. The culture was grown in MSM after inoculation for 10 days with appropriate and uninoculated controls. Quinalphos residue in the culture medium after a 2-day interval was quantified, and results after the subtraction of the values of the control are presented in Table 2.

**Table 2:** Influence of initial concentration of quinalphos degradation by *Bacillus thuringiensis* under submerged culture conditions

Incubation period	Residual concentration of quinalphos in MSM			
Day	20 ppm	50 ppm	100 ppm	200 ppm
0	20	50	100	200
2	3.37 ± 0.06^a^	4.54 ± 0.11^a^	5.50 ± 0.06^a^	6.12 ± 0.06^a^
4	2.30 ± 0.06^b^	3.22 ± 0.06^b^	3.00 ± 0.03^b^	4.75 ± 0.03^b^
6	2.04 ± 0.05^c^	2.37 ± 0.06^c^	2.76 ± 0.06^c^	3.68 ± 0.06^c^
8	0.77 ± 0.09^d^	1.56 ± 0.06^d^	1.55 ± 0.06^d^	2.13 ± 0.04^d^
10	0.53 ± 0.06^e^	1.07 ± 0.04^e^	1.25 ± 0.08^e^	1.64 ± 0.03^e^

Values labelled with the same letter (a, b, c, d, e) are not statistically significant at *P* < 0.05

The concentration of quinalphos had no influence on the degradation of quinalphos by *B. thuringiensis* in this study (Table 2). Degradation of quinalphos occurred to the extent of 75% in the culture of *B. thuringiensis* grown on MSM with the concentration of quinalphos at 20 mg L^−1^ as against 98% in the same culture with 200 mg L^−1^ of quinalphos concentration. No appreciable degradation of quinalphos (1%) was observed in respective inoculated controls in the corresponding period. This observation of the present study is in agreement with the result of degradation of chlorpyrifos at concentration from 10 to 500 mg L^−1^ by *Alcaligenes faecalis* strain DSP3 [32]. Anwar et al. [41] reported degradation of chlorpyrifos by *B. pumilus* C2A1 at even relatively higher concentrations, i.e. 500 and 1000 mg L^−1^, to the extent of 90% after 2 weeks. The prolonged lag phase in the degradation of chlorpyrifos at higher concentration could be due to the time taken for the adaptation of microorganisms to produce the necessary enzymes [42].

### Influence of the medium pH on the biodegradation of quinalphos

**Fig. 5:**
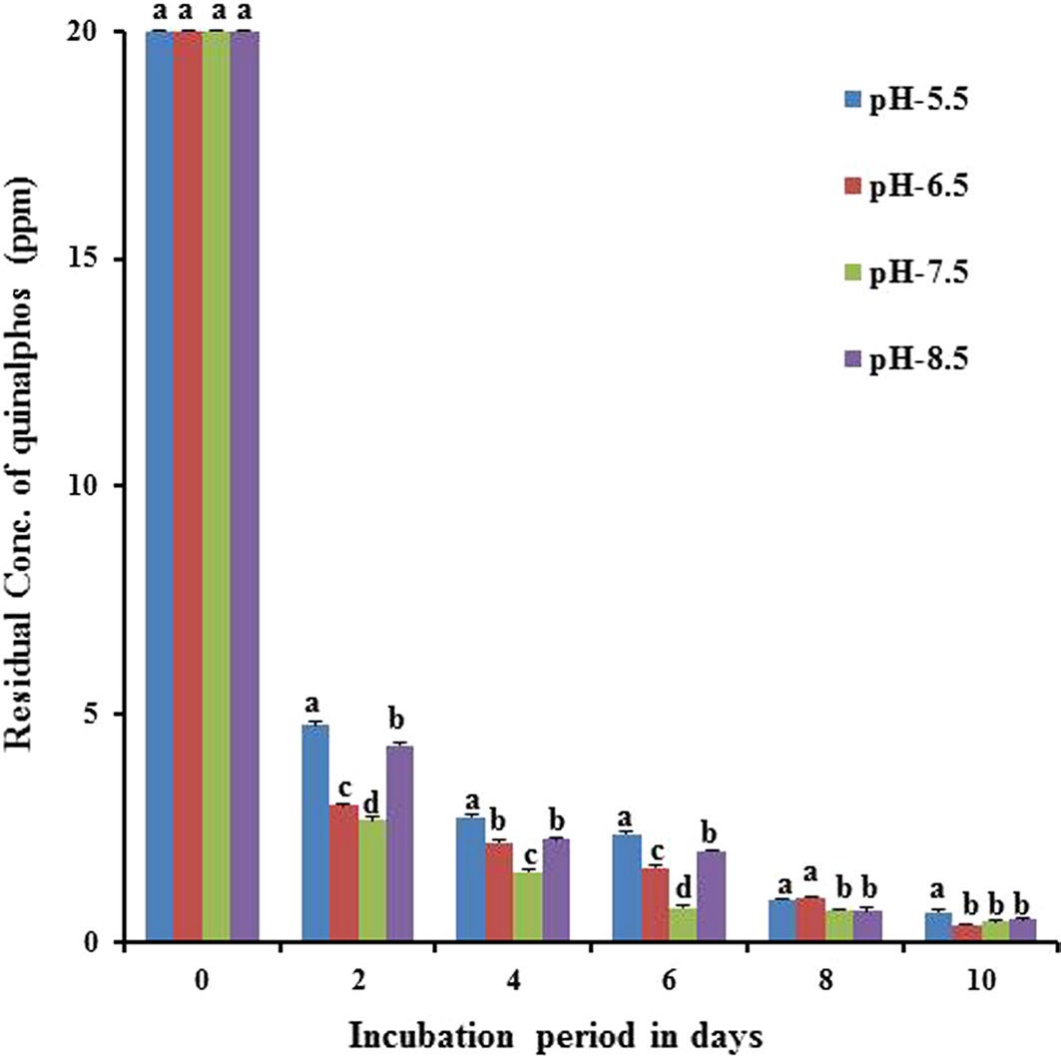
Influence of medium pH on biodegradation of quinalphos. Values represented by *bars* with the same letter (*a, b, c, d*) are not statistically significant at *P* < 0.05

One important factor which influences the degrading ability of microorganisms is pH. It needs to be assessed critically as it greatly determines the survival and activity of the microorganisms. The quinalphos degrading ability of *B. thuringiensis* was studied at different pH conditions, i.e. 5.5, 6.5, 7.5 and 8.5, in MSM fortified with 20 mg L^−1^ quinalphos along with the control (Fig. 5). The potential bacterial strain showed the degradation at all pH conditions with varying degradation percentages. Quinalphos degradation occurred in respect of *B. thuringiensis* to the extent of 76.71, 85.0, 86.55, and 78.95%, which was observed in the culture with an initial pH of 5.5, 6.5, 7.5 and 8.5, respectively, at the end of 2-day incubation. It was clear from the results of the current study that a pH of 6.5–7.5 was optimal for maximum degradation of quinalphos by bacterial strain. Similarly*, Enterobacter* sp. showed a high degradation rate of chlorpyrifos at high pH levels, whereas in acidic conditions, the degradation rate of chlorpyrifos was very slow at an acidic pH [43]. For complete degradation of ethoprophos (an organophosphate pesticide) in a minimal salt medium, two strains of *Pseudomonas putida* (epI and epII) took 48 h at pH levels from 6.3 to 7.6 as against 72 h at pH 5.5 by the same strains [38]. Anwar et al. [41] reported that at an acidic pH level, *Bacillus pumilis* C2A1 showed degradation of nearly 50% of chlorpyrifos with a longer lag phase. At a relatively higher pH level, no lag phase was observed and chlorpyrifos was more efficiently degraded at a basic as well as neutral pH level where by more than 80% of the added pesticide was degraded. According to a recent report by Talwar et al. [23], *Ochrobactrum* sp. strain HZM showed an optimum pH of 7.0 for degradation of quinalphos. This observation is consistent with the result (pH of 7.5 optimal for biodegradation) of the current study. In the current study, maximum degradation was achieved at a high pH. It is possible that some key enzymes responsible for quinalphos degradation have their optimum enzymatic activity in high pH conditions in addition to higher growth.

### Influence of temperature on biodegradation of quinalphos

**Fig. 6:**
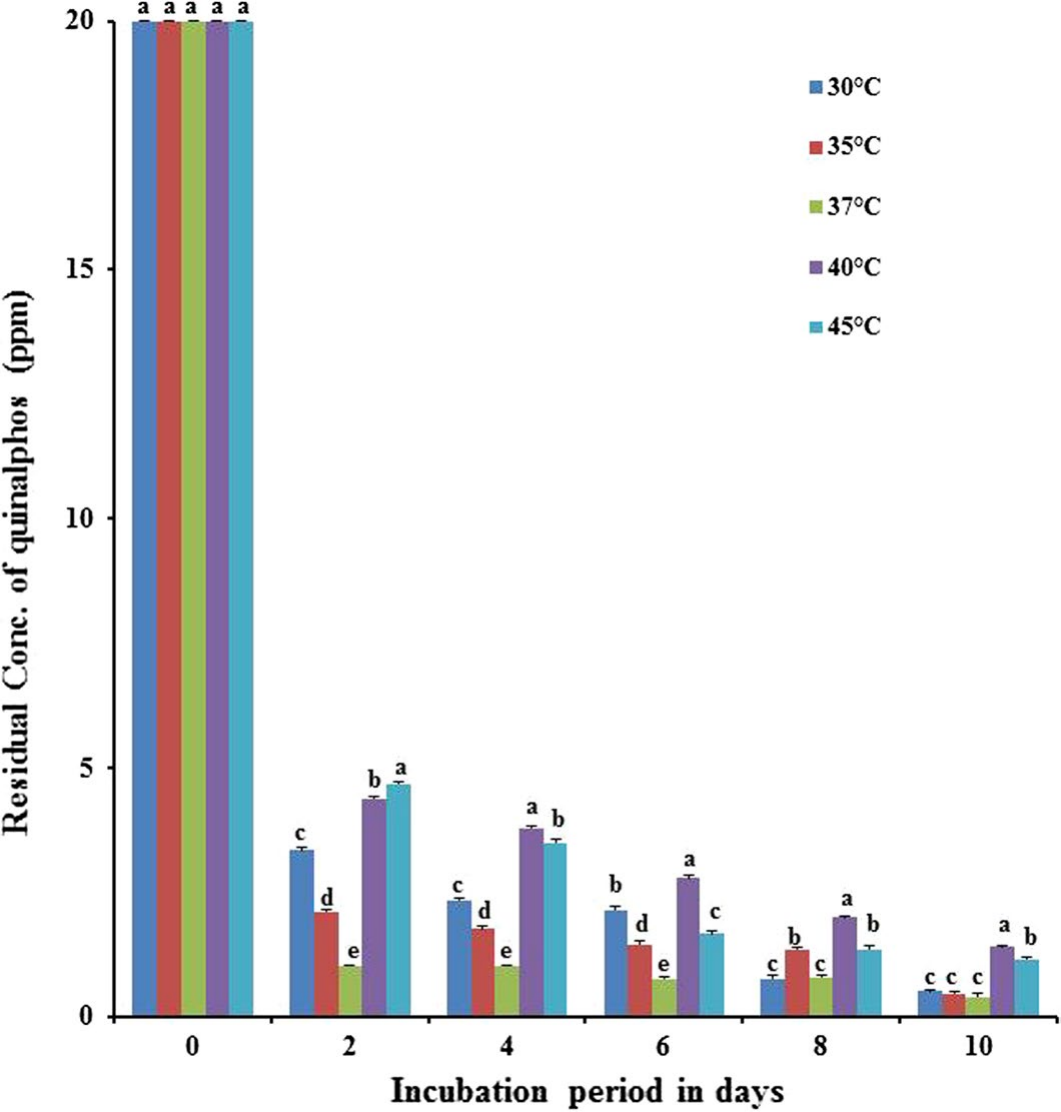
Influence of temperature on biodegradation of quinalphos. Values represented by bars with the same letter (*a, b, c, d, e*) are not statistically significant at *P* < 0.05

In order to find out the optimum temperature for degradation of quinalphos by bacterial isolate *B. thuringiensis*, the culture was cultivated in MSM fortified with 20 mg L^−1^ of quinalphos at different temperatures, i.e. 30, 35, 37, 40 and 45 °C, along with appropriate controls. The bacterial strain *B. thuringiensis* showed the degradation at all temperatures with varying proportions (Fig. 6). *B. thuringiensis* displayed quinalphos degradation to the extent of 83.66, 89.55, 94.74, 77.73 and 76.34% with respective temperatures of 30, 35, 37, 40 and 45 °C at the end of the 2-day incubation. In the current study, the rate of quinalphos by bacterial culture *B. thuringiensis* was low at 30 °C and increased with increasing temperature up to 37 °C and declined thereafter. Based on these results, a temperature within the range of 35–37 °C was optimal for degradation of quinalphos by bacterial strain *B. thuringiensis*. Similarly, the degradation of an organophosphate pesticide, ethoprophos, by two strains of *Pseudomonas putida* (epI and epII) was more rapid at 25 and 37 °C than at other temperatures—5 and 40 °C [38]. According to Singh and Walker [1], maximum degradation rate of fenamiphos and chlorpyrifos by either a BEP consortium or *Enterobacter* sp. was observed at 35 °C. In a recent study, Talwar et al. [23] observed the influence of temperature on degradation of quinalphos by *Ochrobactrum* sp. strain HZM and reported 27 °C for optimal degradation of quinalphos.

## Conclusions

The bacterial isolate from soil samples of a grape vine garden was identified as *B. thuringiensis* through the selective culture enrichment method, morphological, biochemical and 16S rRNA gene sequence analysis. The optimum environmental conditions for growth and degradation of quinalphos were analysed in shaking conditions and recorded as the inoculum density of (1.0. OD), pH (6.5–7.5), 35–37 °C temperature and high concentration of quinalphos (200 ppm). Additional carbon and nitrogen sources (carbon source—sodium acetate) (nitrogen source—yeast extract) marginally improved the rate of degradation of quinalphos. It thus appears that *B. thuringiensis* is the best microbial source to be used in a quinalphos/pesticide-contaminated environment.
